# Levels of oxidative stress in patients with neoadjuvant chemotherapy for gastric cancer: correlation with treatment response

**DOI:** 10.3389/fonc.2023.1192192

**Published:** 2023-05-18

**Authors:** Jiatong Lu, Shaoyu Guan, Jiajun Luo, Jingwen Yuan, Junfeng Yan, Chen Yang, Qiang Tong

**Affiliations:** ^1^ Department of Gastrointestinal Surgery I Section, Renmin Hospital of Wuhan University, Wuhan, China; ^2^ 93868 Troop of the Chinese People's Liberation Army (PLA), Yinchuan, China; ^3^ Department of Gastrointestinal Surgery, The Sixth Hospital of Wuhan, The Affiliated Hospital of Jianghan University, Wuhan, China

**Keywords:** gastric cancer, oxidative stress, neoadjuvant chemotherapy, chemotherapy responsiveness, predictors

## Abstract

**Objective:**

The intent of this study was to investigate the relationship between oxidative stress and treatment response in gastric cancer patients undergoing neoadjuvant chemotherapy.

**Methods:**

Blood samples from 108 patients and 108 healthy subjects were collected, and all patients were enrolled in SOX chemotherapy. The patients received four cycles of neoadjuvant chemotherapy. Blood samples were collected to determine oxidative stress levels at baseline prior to beginning chemotherapy, and at the end of cycles 2 and 4. The patients receiving neoadjuvant chemotherapy were followed up for several months to years. A survival curve was created according to the follow-up information from the patients. In addition, the correlation between oxidative stress level and treatment effect was evaluated and ROC curves were plotted according to the final collected data.

**Results:**

Compared with the normal group, the levels of the antioxidant index decreased while the peroxide index increased in the patients. Conversely, when patients were compared before and after chemotherapy, the antioxidant index increased but the peroxide index decreased. Furthermore, the antioxidant index increased in the response group while the peroxide index decreased in the non-response group.

**Conclusion:**

Patients with an increased antioxidant index after chemotherapy have good treatment responsiveness. These indicators can also be used as predictors to judge the patients’ response to chemotherapy.

## 1 Introduction

Gastric cancer belongs to the category of invasive malignant tumors, and the mortality rate from gastric cancer ranks fourth among cancer deaths, with mortality in Asia and South America being the most common (1, 2). China has one of the highest mortalities of gastric cancer in the world, accounting for 50% of the global deaths due to gastric cancer every year (3). The primary treatment method for gastric cancer is surgery. Chemotherapy is also an indispensable treatment method. For some patients who have metastasis or unresectable tumors, neoadjuvant chemotherapy is crucial. At present, drugs commonly used in chemotherapy for gastric cancer include fluorouracil, oxaliplatin and capecitabine.

Oxidative stress is an unbalanced ratio between the antioxidant capacity and the production and concentration of reactive oxygen species (ROS) in the human body, which tends to oxidize, resulting in inflammatory neutrophil infiltration, increased protease secretion and the production of various oxidative intermediates. Oxidative stress is a major contributor to aging and disease. During oxidative stress, reactive oxygen and nitrogen are accumulated, including nitric oxide (NO), superoxide anionic radicals (O2-), lipid peroxidized radicals (LOO-) and hydroxyl radicals (OH-). Studies have shown that alkylating agents and cisplatin can produce excess free radicals, leading to oxidative stress in cells (4). ROS can also be used as a signaling molecule in cells (5). At the same time, ROS is also deemed as an unavoidable toxic side effect of aerobic metabolism. Excess ROS can damage cellular proteins, lipids and DNA under oxidative stress conditions, eventually leading to cell injury or death (6). ROS can interact with lipids to form malondialdehyde (MDA) (7) which can modify proteins to form protein carbonyls. In addition, the role of NO in oxidative stress is gaining more attention. Studies have shown that superoxide anions can react with NO to form a more cytotoxic peroxynitrous acid (8) that has stronger oxidizing properties and a wider range of functions.

In addition to the oxidation system, the human body also has an antioxidant system to mitigate oxidative stress. The antioxidant system is widely present in human plasma and red blood cells (9), and divided into enzymes and non-enzymes. Key enzymes include glutathione reductase (GPX), peroxidase (POD), catalase (CAT), glutathione S-transferase (GST), superoxide dismutase (SOD), glutathione peroxidase (GSH-PX), tathione reductase (GR) and thioredin peroxidase (TPX) (10). Whereas non-enzymes include vitamin A, vitamin C, vitamin E and uric acid. Glutathione is a tripeptide that is made up of cysteine, glycine and glutamic acid. Due to its active sulfhydryl group (- SH), this protein is prone to dehydrogenation and oxidation. This key feature is what makes it a primary antioxidant in the body. Moreover, GSH is a coenzyme for many enzymes such as GSH-Px, that serve multiple biological processes, including participating in the removal of ROS and protection from oxidative stress (11). Superoxide dismutase (SOD), involved in reactive oxygen species defense, is also an antioxidant enzyme. It can decompose two superoxide anions (O2-) into hydrogen peroxide, which is then catalyzed by catalase and glutathione peroxidase into harmless H2O and O2 (12). SOD is widely present in plant and animal cells, protecting cells ex-posed to oxygen. SOD and CAT serve similar functions. GSH, SOD and CAT are crucial indicators for assessing the body’s antioxidant capacity.

Because the level of oxidative stress in tumor cells is high and ROS is important in tumor progression, changes in oxidative stress indicators during chemotherapy may reflect the efficacy of chemotherapy (13–15). Therefore, we speculate that the level of oxidative stress present during chemotherapy is closely linked to its therapeutic effectiveness.

## 2 Materials and methods

### 2.1 Patients

This study included 108 normal subjects as well as 108 gastric cancer patients, including 56 male patients, 52 female patients, 58 male control cases and 50 female control cases. All patients with gastric cancer received SOX chemotherapy primarily consisting of oxaliplatin and teggio. All patients received blood, liver and kidney function tests; CEA level measurement; chest X-ray; CT; gastroscope and an endoscopic biopsy. A positive gastric cancer diagnosis was given prior to surgical operation. The exclusion criteria were as follows (1): the patient is over the age of 80 or under the age of 18; (2) the patient is not receiving chemoradiotherapy; (3) immunosuppressed patients; (4) patients with a history of chemoradiotherapy; (5) patients with severe infections. All patients obtained and signed informed consent before enrollment, and the Ethics Committee of Renmin Hospital of Wuhan University has approved the study protocol (WDRY2018-K055, 2018.10.31).

### 2.2 Methods

Blood samples were collected from both gastric cancer patients and normal sub-jects at the beginning of neoadjuvant chemotherapy, at the conclusion of cycle 2 and cycle 4. Blood was collected from patients with gastric cancer, coagulated for half an hour, centrifuged (10 minutes, 1000×g) and stored in cryopreservation at -80°C. Serum levels of catalase (CAT), superoxide dismutase (SOD), reduced glutathione (GSH), nitric oxide (NO) and malondialdehyde (MDA) were determined using commercially available kits. (Nanjing Institute of Bioengineering, Nanjing, China). All data was collected and collated in a table. CT examination was conducted at the beginning of neo-adjuvant chemotherapy, and at the end of the second and fourth cycle of chemotherapy.

### 2.3 Statistical analysis

SPSS 22.0 was utilized for data analysis and ROC curve plotting. The experimental results are statistically significant only when the P value is less than 0.05. The biochemical data of the patient and normal subject groups were represented in the form of a mean ± standard deviation and the data were integrated and analyzed. The independent sample t-test was used to compare differences for variables that conform to normal distribution. Using the χ2 test and Fisher exact test for contingency table data that does not conform to normal distribution, the baseline data table and the univariate analysis table were plotted. The statistically significant indicators were represented as ROC curves and the area under the curve (AUC) was calculated. GraphPad Prism 8 was used to plot the survival curves of the response and non-response groups.

## 3 Results

This study included 108 patients and 108 normal healthy subjects. **Table 1** displays the patient-specific data.

**Table 1:** 108 gastric cancer (GC) patients’ characteristics.

Characteristics	Response group (CR+PR) n = 55(%)	Non-response group (PD+SD) n = 53(%)	t/χ2	P
Gender, M	30(54.5)	29(54.7)	0.0003	0.9852
Age, mean±s[years]	63.3±11.2	62.1±10.4	0.5765	0.5655
CT Stage			5.3537	0.0207*
II+III	24(43.6)	16(30.2)		
IV	31(56.4)	37(69.8)		
CN Stage			4.3848	0.0363*
N0	15(27.3)	6(11.3)		
N+	40(72.7)	47(88.7)		
Tumor differentiation			4.1246	0.0423*
Well+Moderately differentiated	26(47.3)	15(28.3)		
Poorly differentiated	29(52.3)	38(71.7)		

*P<0.05.

**Figure 1:**
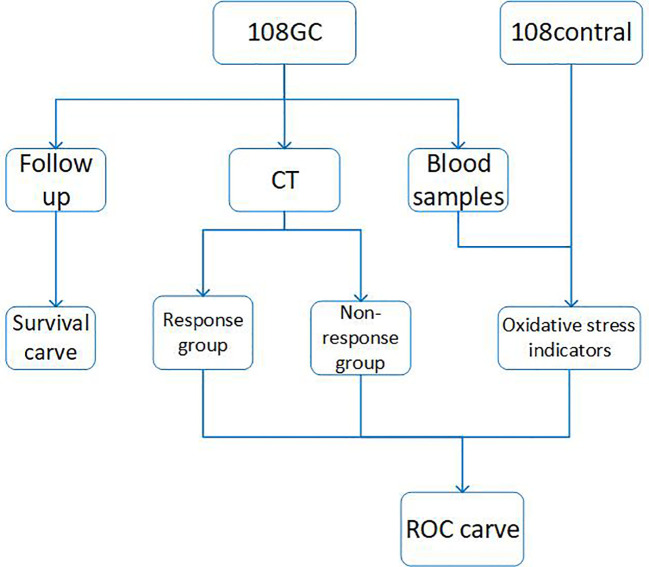
The experimental flowchart of this study.

**Figure 2:**
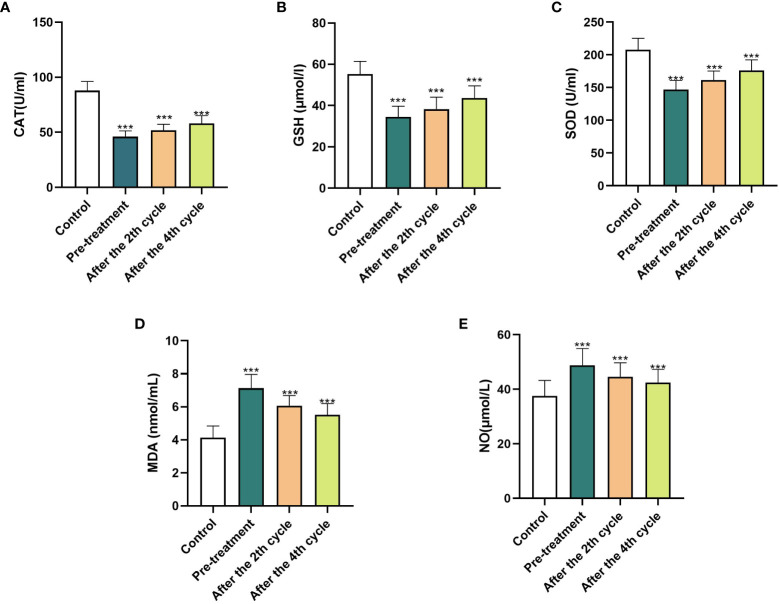
**(A–E)** is the histogram of different oxidative stress indicators. The histogram shows the oxidative stress’s level in each cycle of neoadjuvant chemotherapy in normal control group and gastric cancer patients. Before chemotherapy and after the finish of the 2 and 4 cycles, all the oxidative stress indicators of patients were statistically significant (one-way ANOVA, * * *P<0.001). CAT, catalase; GSH, reducing glutathione; SOD, superoxide dismutase; MDA, malondialdehyde; NO, nitrogen monoxide.

The entire experimental process is shown in **Figure 1**, using commercially available kits, we first measured the levels of CAT, GSH, SOD, MDA and NO in serum from normal control subjects and patients at the beginning of neoadjuvant chemotherapy, at the end of cycles 2 and 4 and then analyzed the data (**Figure 2**). In comparison to the control group, we discovered that CAT, GSH and SOD levels decreased in patients who did not receive neoadjuvant chemotherapy, while MDA and NO levels increased.

In comparison to non-chemotherapy patients, with the progress of chemotherapy, the antioxidant index (CAT, GSH and SOD) in the patients gradually increased and the peroxide index (MA, NO) gradually decreased, but there was still a gap between these and the values of the normal indicators. This shows that the occurrence of cancer is closely associated with the body’s oxidative stress level. The imbalance in oxidative stress levels caused by cancer can be lessened by chemotherapy. Through chemotherapy, the patient’s peroxide level decreased and the antioxidant level increased, but complete recovery may require the combined application of other means. At the end of the four cycles of neoadjuvant chemotherapy, we assessed the number of gastric cancer patients who had a complete response (CR), partial response (PR), progressive disease (PD) and stable disease (SD). All patients received a CT examination before the beginning of chemotherapy and at the conclusion of cycle 2 and cycle 4 to evaluate the curative effect and observe whether the cancer is progressing or receding. The treatment response is classified as follows: CR (16): All target lesions vanish. The short axis of any pathological lymph node (whether target or non-target) must be reduced to < 10 mm; PR: Reduce the total diameter of the target lesion by at least 30%; PD: The sum of the target lesion’s diameter increases by at least 20% or a new lesion appears; SD: The sum of the maximum diameter of the tumor target lesion did not reach PR or the enlargement did not reach PD. PR and CR were co-classified into the response group, and PD and SD were co-classified into the non-response group. **Tables 2**, **3** show the levels of oxidation and antioxidant indicators in the response and non-response groups, respectively. GSH, SOD, CAT, NO and MDA levels were measured in both groups. In comparison to the non-respondent group, we found that the response group had significantly higher levels of CAT, GSH and SOD following the conclusion of the second and fourth cycle, while the levels of MDA and NO decreased. We also found that the effectiveness of neoadjuvant chemotherapy was related to the TNM stage and pathological grade of cancer, but not related to age or gender factors.

**Table 2:** Comparison of oxidant levels in the response (CR+PR) and non-response (SD + PD) groups.

	MDA (nmol/mL) mean ± SD			NO (μmol/L) mean ± SD		
Controls (n = 108)	4.13±0.71			37.53±5.72		
Cancer patients (n = 108)	Response group	Non-response group	P value	Response group	Non-response group	P value
Pre-treatment	7.32±0.88	6.94±0.75	<0.001***	50.22±6.32	47.23±5.66	0.011*
After the 2 cycles	5.73±0.47	6.42±0.54	<0.001***	43.83±4.35	45.33±5.75	0.012*
After the 4 cycles	5.12±0.43	5.93±0.63	<0.001***	41.25±3.78	43.75±5.39	0.006*

*P<0.05, ***P<0.001.

CR, complete response; PR, partial response; PD, progressive disease; SD, stable disease.

**Table 3:** Comparison of antioxidant levels in the response group (CR+PR) and non-response group (SD + PD).

	GSH (μmol/l) mean± SD			SOD (U/ml) mean ± SD			CAT(U/ml) mean ± SD		
Controls (n = 108)	55.24±6.22			207.73±17.58			87.96±8.25		
Cancer patients (n = 108)	Response group	Non-response group	P value	Response group	Non-response group	P value	Response group	Non-response group	P value
Pre-treatment	35.43±5.23	33.69±4.87	0.076	150.65±12.23	142.89±14.77	0.004*	46.73±5.61	45.39±4.69	0.182
After the 2 cycles	40.32±5.71	36.15±5.12	<0.001***	166.45±13.37	155.93±12.69	<0.001*	53.19±5.53	50.63±4.89	0.013*
After the 4 cycles	45.77±4.37	40.42±5.62	<0.001***	184.31±12.56	184.31±15.32	<0.001*	61.59±6.87	54.39±5.21	<0.001*

*P<0.05, ***P<0.001.

CR: complete response PR: partial response PD: progressive disease SD: stable disease

**Figure 3:**
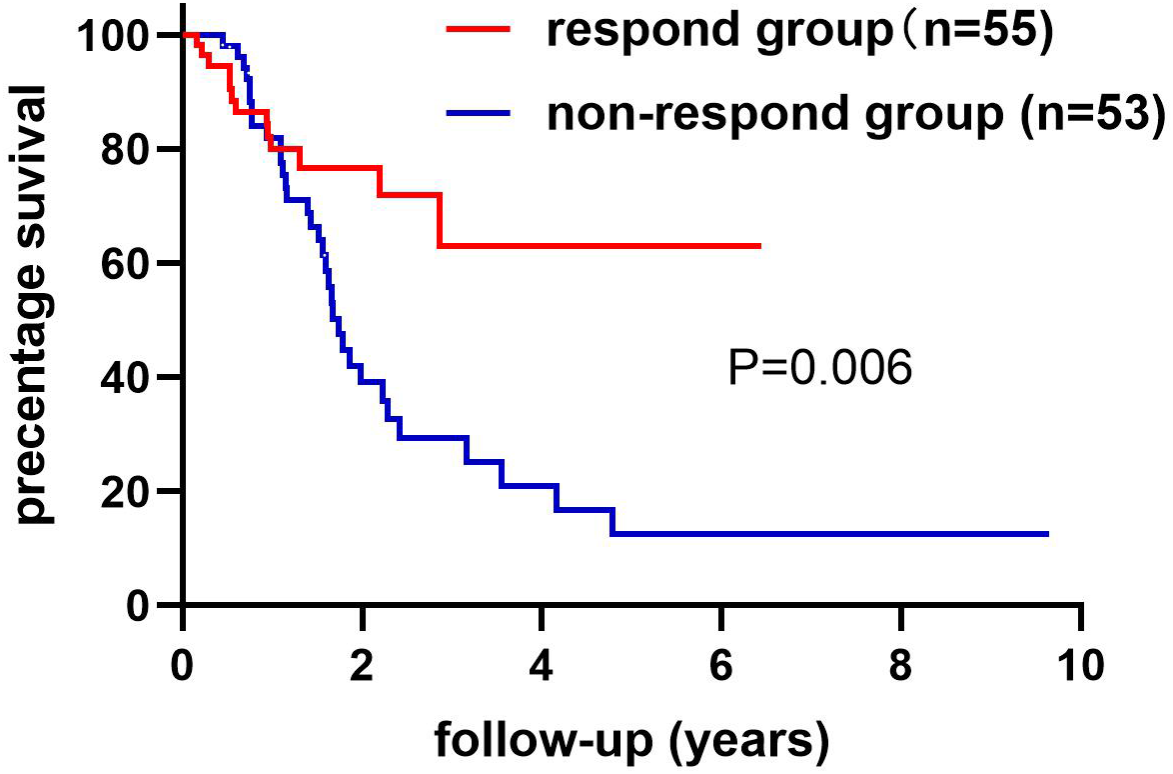
The non-response and response groups’ follow-up ended with death or loss of connection. The response group had 55 cases and the non-response group had 53 cases. The difference in survival rates between the response and non-response groups has statistical significance. Log-rank (Mantel-Cox) text. P=0.006.

**Figure 4:**
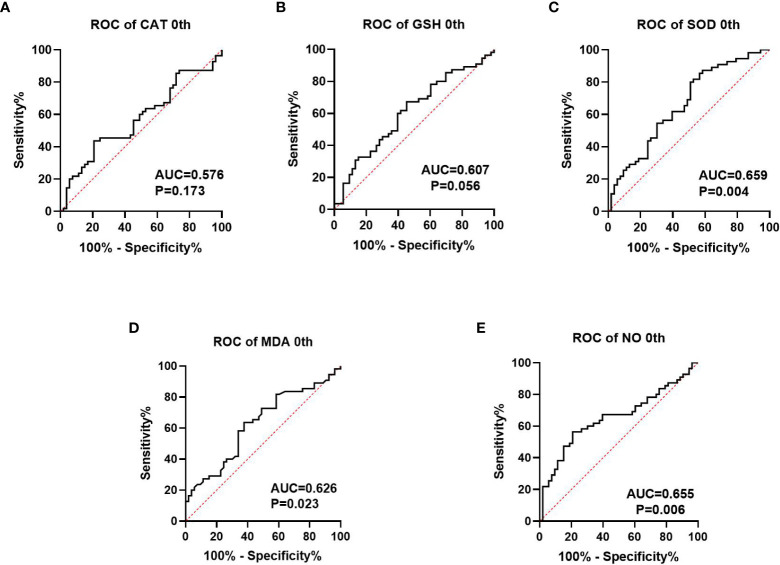
Analysis of ROC curve of the oxidative stress index in patients prior to neoadjuvant chemotherapy, where **(A–C)** represents the antioxidant oxidation index and **(D, E)** represents the peroxide index. P<0.05 has statistical significance. **Figures 3A**, **B** have no statistical significance.

**Figure 5:**
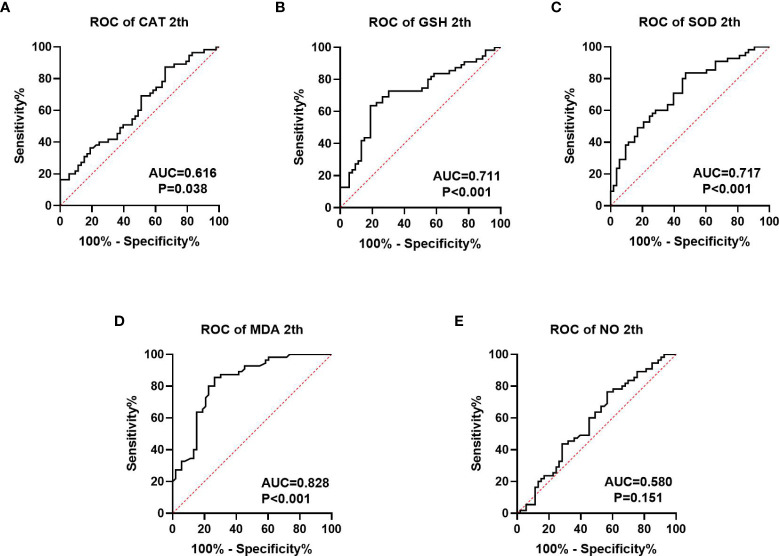
ROC curve analysis of oxidative stress index in patients after 2 cycles of neoadjuvant chemotherapy, **(A–C)** is antioxidant oxidation index, **(D, E)** is peroxide index. P<0.05 has statistical significance. **Figure 4E** has no statistical significance.

**Figure 6:**
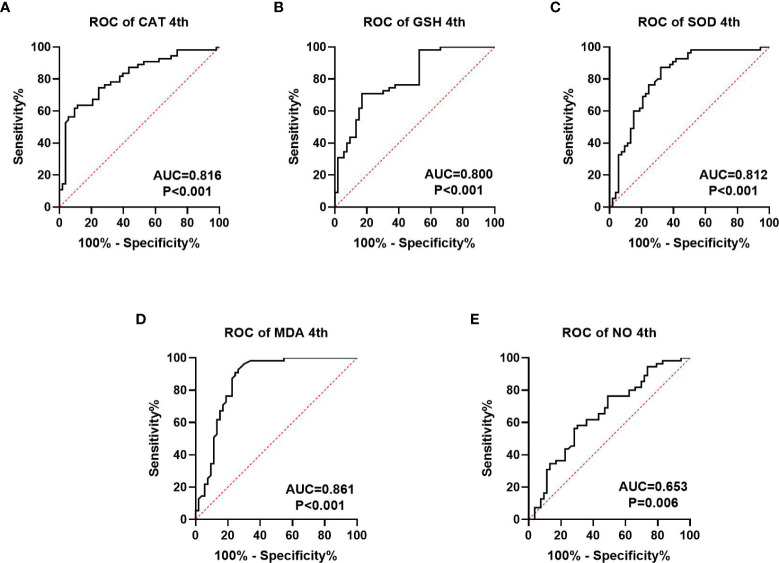
ROC curve analysis of oxidative stress index in patients after four cycles of neoadjuvant chemotherapy, **(A–C)** is antioxidant oxidation index, **(D, E)** is peroxide index. P<0.05 is statistically significant.

We collected information on patient outcomes after four cycles of neoadjuvant chemotherapy and plotted survival curves (**Figure 3**). The response group had a longer survival time than the non-response group. It can be seen from the above table that CAT, GSH, SOD, MDA and NO levels were all statistically significant. All indicators with statistical differences were drawn on the ROC curve (**Figures 4**–**6**) and the AUC measured. Before the beginning of neoadjuvant chemotherapy, the values of CAT, SOD, MDA and NO were statistically significant, while the values of GSH were not statistically significant, with AUC values of 0.576, 0.659, 0.626 and 0.655. At the end of two cycles, the values of CAT, GSH, SOD and MDA were statistically significant, while the values of NO were not statistically significant, with AUC values of 0.616, 0.711, 0.717 and 0.828. At the completion of four cycles, all indicators were statistically significant. The values of CAT, GSH, SOD, MDA and NO were 0.816, 0.800, 0.812, 0.861 and 0.653. This indicates that the level of oxidative stress indicators can be used as a predictor of the responsiveness of patients to chemotherapy. With the gradual extension of chemotherapy time, its accuracy will slowly improve.

## 4 Discussion

Gastric cancer has remained a significant malignant cancer with limited early detection and high mortality (17). Successful treatment has become a major problem to overcome in the clinic. With technical advances in surgery, radiotherapy and chemotherapy, the survival rate of gastric cancer patients has greatly improved (18). If patients with gastric cancer are diagnosed earlier or treated before the cancer cells spread, patients’ survival rate is 60%; if the cancer cells have invaded other deep tissues, patients’ survival rate is reduced to 31%. Patients’ survival rate drops to 5% if cancer cells migrate to distant areas of the body (19, 20). Chemotherapy drugs for gastric cancer generally consist of fluorouracil, oxaliplatin and other drugs, but due to some individual differences between patients (21), the effect of drug chemotherapy varies from person to person (22). Therefore, observing the treatment responsiveness of patients in the early stage of chemotherapy will help establish patient confidence and guide the design and adjustment of treatment plans.

In many cancers, oxidative stress levels will increase gradually as the disease progresses (23, 24). Studies have shown that oxidative stress levels in tumor tissues are significantly increased after chemotherapy or radiotherapy, and the elevated reactive oxygen radicals can persist in the microenvironment for months or even years (25, 26). GSH is an important lipid peroxide reductase that can reduce toxic and carcinogenic effects on cells by removing ROS from the human body (27). CAT and SOD are also enzymes that cells use to fight peroxidation states, and studies have shown that glutathione depletion can make cancer cells more sensitive to oxidative stress and thereby more susceptible to some pro-apoptotic genes or anti-cancer drugs (28). MDA is produced by lipid peroxidation, which has toxic effects on normal cells and is a known tumor promoter and carcinogenic factor (29).

This study measured the levels of the oxidative stress indicators GSH, SOD, CAT, MDA and NO in the serum of neoadjuvant chemotherapy patients and explored their relationship with treatment responsiveness. We found that, in those with gastric cancer who were not given neoadjuvant chemotherapy, the levels of CAT, GSH and SOD were significantly lower than in the control group, while the levels of MDA and NO increased significantly. In comparison to those who didn’t get chemotherapy, in those receiving neoadjuvant chemotherapy, the antioxidant levels began to recover (CAT, GSH and SOD increased) and the peroxide level decreased (MDA and NO decreased). This indicates that chemotherapeutic drugs can antagonize the peroxidation state produced by tumor cells. We divided chemotherapy patients into response as well as non-response groups and compared the two groups’ levels of oxidative stress. The results show that in the response group, GSH, SOD and CAT levels increased while MDA and NO levels decreased when compared to the non-response group. To sum up, we conclude that chemotherapy drugs can change the level of oxidative stress by killing tumor cells. When the antioxidant index in the body increases and the peroxide index decreases, it means that chemotherapy drugs have higher sensitivity to tumors, this also indirectly indicates that the oxidative stress index can be used as a predictor of the degree of chemotherapy response. An increase in peroxide levels indicates damage to tissues and cells (30) finally leading to reduced sensitivity to drug chemotherapy. Some articles have shown that GSH (31) and SOD (32) play an antioxidant role in cells. In the cases of non-small cell lung cancer, as well as, cervical cancer (33), the response group has higher levels of GSH and SOD in their serum than those who do not respond to chemotherapy, which is the same as our experimental results. MDA and NO can reflect the peroxidation state of cells (34). Shatzer et al. (35) have shown that ROS could inhibit the efficacy of chemotherapy drugs such as cisplatin on cancer cells. Oxidative stress products also inhibit cell cycle progression causing blockade of cell cycle checkpoints, and ultimately interfering with the anticancer drugs’ ability to kill cancer cells (9). Therefore, the level of oxidative stress may be linked to the patient’s response to neoadjuvant chemotherapy.

At present, the common biomarkers of postoperative chemotherapy sensitivity for gastric cancer and colorectal cancer are CA724 (36, 37), CA199 and CEA (38). We plotted ROC curves of GSH, SOD, CAT, MDA and NO at different chemotherapy stages and computed the area under the curve. Before the beginning of neoadjuvant chemotherapy, the values of CAT, SOD, MD and NO were statistically significant, while the value of GSH was not statistically significant, the AUC values are 0.576, 0.659, 0.626, and 0.655. At the end of two chemotherapy cycles, the values of CAT, GSH, SOD and MDA were statistically significant, the AUC values were 0.616, 0.711, 0.717 and 0.828. While the value of NO was not statistically significant. Following the completion of the fourth chemotherapy cycle, all indicators were statistically significant. The values of CAT, GSH, SOD, MDA and NO were 0.816, 0.800, 0.812, 0.861 and 0.653, respectively. This indicates that the oxidative stress index may be used as a predictive index of neoadjuvant chemotherapy for patients and may also have a predictive effect on the efficacy as well as prognosis of those receiving postoperative chemotherapy. However, this report studies the predictive level of a single index for neoadjuvant chemotherapy. Whether the combined measurement of multiple indicators can further reflect the efficacy of chemotherapy remains to be further explored.

GSH, SOD, CAT, MDA and NO are commonly utilized oxidative stress indicators in the clinic. In the study, these indicators could be used to assess the patients’ therapeutic response to chemotherapy and could also be used as predictors of treatment responsiveness to neoadjuvant chemotherapy. However, our experiment still has many limitations, we only measured oxidative stress indicators in serum and did not test erythrocytes for oxidative stress. In addition, different chemotherapy schemes may also have different effects on the levels of oxidative stress indicators. We also only measured the oxidative stress level in patients under one chemotherapy regimen, in the future, the experimental design needs to be expanded.

## 5 Conclusion

We conclude that when the antioxidant system in the patient is active or the peroxidation system is inactive, gastric cancer patients have high therapeutic responsiveness to neoadjuvant chemotherapy. When peroxidation in the body is high or the antioxidant capacity is low, the chemo-responsiveness of patients with neoadjuvant chemotherapy is reduced. GSH, SOD, CAT, MDA and NO may also be used to predict the responsiveness of chemotherapy in patients. These findings have high clinical ap-plication value.

## Data availability statement

The raw data supporting the conclusions of this article will be made available by the authors, without undue reservation.

## Ethics statement

The studies involving human participants were reviewed and approved by The Ethics Committee of Renmin Hospital of Wuhan University has approved the study protocol (WDRY2018-K055, 2018.10.31). It is affiliated with Renmin Hospital of Wuhan University. The patients/participants provided their written informed consent to participate in this study.

## Author contributions

QT designed the research and revised the manuscript. JTL, SG, JJL, JWL, JFY and CY added the details of the research. JTL analyzed the research results as well as wrote the manuscript. SG, JJL and JTL conducted experiments. JWY, JFY and CY collected and verified data. The ultimate manuscript had been read and approved by all of the authors
